# Understanding the Medico-Legal Aspects of Telemedicine in India

**DOI:** 10.7759/cureus.42431

**Published:** 2023-07-25

**Authors:** Satvik N Pai, Madhan Jeyaraman, Naveen Jeyaraman, Sankalp Yadav

**Affiliations:** 1 Orthopaedic Surgery, HOSMAT Hospital, Bangalore, IND; 2 Orthopaedic Surgery, Sri Ramachandra Institute of Higher Education and Research, Chennai, IND; 3 Orthopaedics, South Texas Orthopaedic Research Institute (STORI), Laredo, USA; 4 Orthopaedics, ACS Medical College and Hospital, Dr MGR Educational and Research Institute, Chennai, IND; 5 Medicine, Shri Madan Lal Khurana Chest Clinic, New Delhi, IND

**Keywords:** law, litigation, india, medicolegal, telemedicine

## Abstract

Telemedicine has brought a new dimension to healthcare and has been gaining popularity worldwide. However, the medico-legal aspects of the practice of telemedicine in India remain ambiguous to most doctors and administrators. We have therefore provided a concise overview of the legal aspects of telemedicine, which will enable doctors and administrators to provide better telemedicine services while safeguarding themselves from possible litigation.

## Editorial

Telemedicine has been defined by the World Health Organization as "the delivery of health care services using information and communication technologies for the exchange of valid information for diagnosis, treatment, and prevention of disease and injuries, research and evaluation, and for the continuing education of health care providers, where distance is a critical factor" [1]. Telemedicine has brought a new dimension to healthcare. While telemedicine has seen a steadily growing trend over the last decade, it has seen an unforeseen rise in users since 2020 due to the COVID pandemic. Telemedicine has several benefits, including increased access to care, lower overall costs, and high patient satisfaction rates [2]. It, however, raises issues pertaining to professional liability, confidentiality, credentialing, reimbursement, and the development of standards and infrastructure. Telemedicine has been gaining popularity in India too. However, the medico-legal aspects of the practice of telemedicine in India remain ambiguous to most doctors and administrators.

The law

There is currently no law in India specifically pertaining to telemedicine. While the Information Technology Act of 2000 dealt with information technology, it did not address issues in relation to healthcare. This has led several people to question the legality of telemedicine in India for decades. However, the absence of any law specifically for telemedicine should not be interpreted to mean that the practice of telemedicine is illegal in India. In March 2020, the Government of India released the ‘Telemedicine Practice Guidelines’ which have been incorporated as Appendix 5 of the Indian Medical Council (Professional Conduct, Etiquette, and Ethics) Regulation, 2002 [3].

This provides a framework for the practice of telemedicine. Some of the salient features of the guidelines are as follows:

Who can practice telemedicine?: Any Registered Medical Practitioner (RMP) enrolled in the State Medical Register or the Indian Medical Register is permitted to provide telemedicine consultations. No special qualifications are required at present. The government, however, plans to introduce an online program in the future that will require completion to enable a RMP to practice telemedicine.

Identification of RMP, patient: Teleconsultations are not permitted to be anonymous. The RMP must identify himself along with his qualifications at the beginning of the consultation. The RMP must also explicitly verify the name, age, and identification of the patient.

Mode of telemedicine to be used: This has been left to the discretion of the RMP based on the complaints, the need for visual or audio interaction, the condition of the patient, and the available resources. If the RMP feels an in-person consultation is warranted, he reserves the right to recommend the same and proceed only after an in-person consultation.

Consent: If a patient initiates the teleconsultation, consent is implied. If the RMP initiates a teleconsultation, consent has to be obtained from the patient. This explicit consent by the patient can be in the form of an email, a text message, or stating his intent over the phone or video.

Prescribing medicines: It involves the same level of professional responsibility as face-to-face consultations. Prescribing medications without a provisional or appropriate diagnosis may be considered professional misconduct. Under telemedicine, the RMP (Registered Medical Practitioner) can prescribe certain medications, including common 'over-the-counter' medicines (list O drugs), relatively safe drugs with low abuse potential for first-time prescriptions (list A drugs), or for refills (list B drugs). However, the prescription of medicines listed in Schedule X of the Drug and Cosmetic Act and Rules or any Narcotic and Psychotropic substances listed in the Narcotic Drugs and Psychotropic Substances Act, 1985, is strictly prohibited (Prohibited List Drugs). The prescriptions must adhere to the standard rules outlined in the Indian Medical Council (Professional Conduct, Etiquette, and Ethics) Regulations. Furthermore, every prescription must include the RMP's medical council registration number. The RMP is required to provide a photo, scan, digital copy of a signed prescription, or an e-prescription to the patient via email or any messaging platform [3].

Ethics and confidentiality: RMPs must adhere to the same principles of medical ethics, which include following professional standards to safeguard patient privacy and confidentiality, as stated in the IMC (Indian Medical Council) Act. This requirement applies not only to their clinical practice but also extends to their use of technological services. RMPs should exercise a reasonable level of caution when engaging in technological services to prevent any violation of patient privacy or confidentiality [3].

Misconduct: Engaging in telemedicine against a patient's preference to visit a facility or have an in-person consultation, misusing patient images and data, or promoting telemedicine through advertisements would all be considered misconduct for RMPs. The penalties for such offenses will be in accordance with the IMC Act, professional ethics, and relevant laws, similar to any other form of professional misconduct [3].

Documentation: It is the responsibility of the RMP to maintain a record of the teleconsultation, patient records, and prescription records as required for in-person consultations.

Fees: RMP can charge an appropriate fee for the services. He has to provide a receipt or invoice for the fee charged to the patient.

Emergencies: In the event of a medical emergency, the patient must be advised to have an in-person interaction with a RMP at the earliest. Guidance for first aid, counseling, and referrals are to be facilitated.

How the courts look at it

The fact that the ‘Telemedicine Practice Guidelines’ are guidelines and not laws does not mean they are not legally binding. The judiciary and legislature recognize the nascent nature of legal issues pertaining to telemedicine and have therefore provided the framework in terms of ‘guidelines’ that need to be followed until further legislation and laws are formulated and passed. At this time, there have been only a handful of case judgments related to telemedicine in Indian Courts.

In the case of Deepa Sanjeev Pawaskar and Another Vs State of Maharashtra, a postnatal woman visited the hospital where she had delivered a day earlier, with complaints of severe vomiting. She was examined only by a nurse and admitted. The doctor advised for medicines through a telephone conversation with the nurse [4]. Treatment advice was given only through telephone conversation. The patient’s condition later deteriorated, and the patient unfortunately passed away, with the cause of death being determined to be pulmonary embolism. The Bombay High Court held the doctor negligent for having prescribed medicines through the telephone without having gained all the necessary medical information and for prescribing medicine without arriving at a diagnosis.

In the recent high-profile case involving the death of late actor Sushanth Singh Rajput, the prescription of clonazepam to him by a doctor came under contention in court after it was claimed that the drug was procured through a teleconsultation [5]. However, with clonazepam being listed on the prohibited list, the alleged procurement of the drug was deemed illegal, citing the Telemedicine Practice Guidelines.

To conclude, the legal framework for telemedicine in India is still evolving. The latest guidelines for 2020 still do not address matters relating to the use of remotely operated invasive or surgical procedures or the use of telemedicine for research purposes. It also does not specify its applicability for consultations with RMPs or patients outside the jurisdiction of India. With the increasing use of telemedicine, the laws guiding it will need to evolve and become more robust as well.
